# Single and Multi-Strain Probiotics Supplementation in Commercially Prominent Finfish Aquaculture: Review of the Current Knowledge

**DOI:** 10.4014/jmb.2202.02032

**Published:** 2022-06-07

**Authors:** Md Afsar Ahmed Sumon, Tofael Ahmed Sumon, Md. Ashraf Hussain, Su-Jeong Lee, Won Je Jang, S. M. Sharifuzzaman, Christopher L. Brown, Eun-Woo Lee, Md. Tawheed Hasan

**Affiliations:** 1 Department of Marine Biology, King Abdulaziz University, Jeddah-21589, Saudi Arabia; 2 Department of Fish Health Management, Sylhet Agricultural University, Sylhet-3100, Bangladesh; 3 Department of Fisheries Technology and Quality Control, Sylhet Agricultural University, Sylhet-3100, Bangladesh; 4 Biopharmaceutical Engineering Major, Division of Applied Bioengineering, Dong-Eui University, Busan 47340, Republic of Korea; 5 Department of Biotechnology, Pukyong National University, Busan 48513, Republic of Korea; 6 Institute of Marine Sciences, University of Chittagong, Chittagong 4331, Bangladesh; 7 FAO World Fisheries University Pilot Programme, Pukyong National University, Busan 48513, Republic of Korea; 8 Core-Facility Center for Tissue Regeneration, Dong-Eui University, Busan 47340, Republic of Korea; 9 Department of Aquaculture, Sylhet Agricultural University, Sylhet-3100, Bangladesh

**Keywords:** Probiotics, growth, immunity, immune-related gene, disease resistances

## Abstract

The Nile tilapia *Oreochromis niloticus*, Atlantic salmon *Salmo salar*, rainbow trout *Oncorhynchus mykiss*, olive flounder *Paralichthys olivaceus*, common carp *Cyprinus carpio*, grass carp *Ctenopharyngodon idella* and rohu carp *Labeo rohita* are farmed commercially worldwide. Production of these important finfishes is rapidly expanding, and intensive culture practices can lead to stress in fish, often reducing resistance to infectious diseases. Antibiotics and other drugs are routinely used for the treatment of diseases and sometimes applied preventatively to combat microbial pathogens. This strategy is responsible for the emergence and spread of antimicrobial resistance, mass killing of environmental/beneficial bacteria, and residual effects in humans. As an alternative, the administration of probiotics has gained acceptance for disease control in aquaculture. Probiotics have been found to improve growth, feed utilization, immunological status, disease resistance, and to promote transcriptomic profiles and internal microbial balance of host organisms. The present review discusses the effects of single and multi-strain probiotics on growth, immunity, heamato-biochemical parameters, and disease resistance of the above-mentioned finfishes. The application and outcome of probiotics in the field or open pond system, gaps in existing knowledge, and issues worthy of further research are also highlighted.

## Introduction

Aquaculture, the farming of aquatic organisms under controlled environments, is a diverse food producing activity growing annually by 4.5%, accounting for a value of USD 243.26 billion [1]. Data suggest that fish, macro-algae, molluscs and crustaceans are meeting the protein demand of an increasing global population, contributing 49.1, 27.3, 15.6, and 7.9%, respectively, to total aquaculture production, equalling ~20% of total global animal protein supplies [2]. Aquaculture practices have shifted from extensive to super-intensive to elevate production at the expense of huge amounts of artificial feeds and degradation of aquatic environments. Poor culture management causes immunosuppression, stress and creates suitable conditions for the proliferation of opportunistic microbial pathogens (bacteria, viruses, and parasites) leading to infectious disease outbreaks. Hemorrhage, exophthalmia, meningoencephalitis, anorexia, and disruption of the nervous system are common impacts of infectious diseases responsible for high mortality of in cultured fishes. To control diseases, antibiotics, drugs and vaccines are routinely used in aquaculture [3].

Vaccination is a well-established strategy for long-term disease protection, but it has been associated with temporary loss of appetite and growth, intra-abdominal lesions and impaired antibody production in fish [4]. On the other hand, excessive use of antibiotics results in the development of antibiotic resistant pathogens [5, 6], immunosuppression in host and reduction or mass killing of normal microbiota in the culture environment and gut [7], including residual effects in human [8]. The magnitude of this problem can be severe; an estimated 1.7 million deaths were attributed to antibiotic resistant bacteria in 2019 [9]. Antibiotic-resistant pathogens have been isolated from fish and humans, and their applications in aquaculture are under strict regulation [2]. To address this concern, one productive line of research has focused on biologically benign approaches such as probiotics, prebiotics, immunostimulants and functional feeds that enhance host immunity to pathogens [10, 11].

The word “probiotics” means “for life” and is based on the Greek word’s “pro” and “bios”. Probiotics were first defined by Parker [12] as “organisms or substances engagement for the maintenance of the microbial balance in the intestine”. These definitions were revised by Merrifield *et al*. [13] for aquatic organisms as, “a probiotic organism can be regarded as a live, dead or component of a microbial cell, which can be administered via feed or into rearing water, benefiting the host by improving growth performance, feed utilization, immune health status, infectious disease resistance, and stress responses which is achieved at least in part via improving the microbial balance in hosts or ambient environment”. Probiotic research in the last decades has revealed positive effects on growth [14, 15], digestive enzymes activities and feed utilization [16], immunity with upregulation of immune related genes [17, 18], improvement of beneficial microbes in the gut and positive modification of intestinal structure [19], and disease protection [20, 21], leading to an eco-friendly aquatic environmental management [22] in fish and shellfish farming.

Previous reviews of probiotics in aquaculture have considered sustainability issues [3], carp culture [7], disease control [2], mitigation of challenges in aquaculture [23], uses in Chinese aquaculture [5] and so on. There is not a single review that has comprehensively addressed the effects of probiotics on prominent cultured finfishes (*i.e.*, tilapia *Oreochromis niloticus*, Atlantic salmon *Salmo salar*, rainbow trout *Oncorhynchus mykiss*, olive flounder *Paralichthys olivaceus*, common carp *Cyprinus carpio*, grass carp *Ctenopharyngodon idella* and rohu carp *Labeo rohita*) as relating to growth, haemato-immunological and disease responses. The novelty of the present review is to present up-to-date insights on the use of probiotics in commercially important finfish culture, highlighting their applications in field or open pond system. Probiotics effects on the host transcriptome profiles and aquatic environmental parameters are also discussed.

## Probiotics Functionality Linked to Growth, and Cellular and Humoral Immunity

Probiotics not only reduce intestinal pathogens but also detoxify harmful compounds in feed through hydrolytic enzymes and the production of vitamins, biotin, and vitamin B12 [24, 25]. Growth elevation with improved feed utilization can ensure improved yields at lower production costs which are of value to aquaculture. Several authors have characterized responses to probiotics including alterations of digestive enzyme synthesis and release [26] and of intestinal structure to increase nutrient digestion and absorption [27], influencing the final body weight gain and production scale.

In aquaculture, probiotics can be supplemented through feed or water to manipulate the microbial balance in host and culture environment. Different nutrient specific digestive enzymes (*e.g.*, amylase, protease, chitinase and lipase) can be increased by probiotics in the intestine [28], improving digestion and absorption while removing toxicity. Jang *et al*. [29] mentioned that probiotics improved digestive process by enhancing beneficial bacterial population, microbial enzymatic activity, and microbial balance, ultimately improving digestibility and absorption of nutrients toward increased growth rates and diet digestibility. It is unknown whether digestive enzymes are produced directly by probiotics or by modulating synthesis and secretion of enzymes by intestinal cells, or possibly by both mechanisms. However, probiotics reportedly demonstrate in vitro extracellular amylase, cellulase, lipase and protease production and could potentially modulate those enzymatic functions in vivo [30].

Normal light microscopic observation on fish gut histology depicts an intact epithelium barrier, goblet cells, and well organised villi and microvilli. Supplemented probiotics metabolize the carbohydrates (oligosaccharides/prebiotics) of diet for their growth and survival in the intestine. A combined application of probiotics and prebiotics, termed synbiotics, produced significantly higher effects compared to each individual component [11, 31]. Fermentation of prebiotics by probiotics is reported to produce short chain fatty acids, which can be utilized as an energy source by intestinal epithelial cells [32], and cell proliferation ultimately increases goblet cell intensity, villus and micro villus height, width and density [33]. Alteration of these morphometric structures also can be found in intestinal thickness, fold of change, and mucus layer, contributing to the elevation of nutrient absorption. Higher nutrient absorption through increased absorption area in the intestine in response to probiotics supplementation showed positive changes in apparent digestibility coefficient [19] that correlated with fish growth and utilization of diets.

Probiotics mainly upregulate the fish innate immune parameters through antigen presenting dendritic cells (DCs) to maintain the linkage between innate and adaptive immunity [34]. Mucus on body surface, scale, skin, and gills serve as physical or epithelial barriers as a first line of defence in fish innate immune system. The role of probiotics to improve epithelial barrier is not well defined, but immunoglobulins (Ig), antibacterial peptides and complement proteins containing skin mucus lysozyme activity were enhanced after probiotics supplementation [35, 36]. Macrophage and phagocytic cells (natural killer cell, neutrophil, monocyte, lymphocyte, and cytotoxic cells) provide cellular immune defence in fish [37]. Interaction between microbial-associated molecular patterns (MAMPs) and pattern recognition receptors (PRRs) on DCs can stimulate and activate the phagocytic cells or increased production of cytokines [38]. Lactic acid bacteria (LAB), for examples *Bacillus* and bifidobacteria are commonly used gram-positive probiotics, and their cell wall components like peptidoglycans, lipopolysaccharides, flagella, and microbial nucleic acids are collectively known as MAMPs. MAMPs are attracted by PRRs on DCs and after interaction/binding of MAMPS with PRRs, DCs stimulate and activate phagocytic cells [39] to locate and engulf invading pathogens. Stimulation of plasma cells by DCs produces antibodies which can pass through the enterocyte and neutralize the pathogens [8]. Moreover, binding of MAMPs with Toll-like receptors (TLR) and stimulation of T cells by DCs produce mostly pro-inflammatory cytokines [31]. Among different pro-inflammatory cytokines, tumor necrosis factor (TNF)-α stimulate neutrophil base immunity [40], interleukin (IL)-1β proliferate lymphocyte and macrophage [41], interferon (IFN)-γ stimulate resting macrophages to secrete IL-6 and IL-1β, and multi-functional IL-6 are responsible for B-lymphocyte differentiation and maturation [42] to elevate cellular innate immunity.

Humoral immunity refers to activities of bacteriolytic or haemolytic enzymes like myeloperoxidase or peroxidase, serum/skin mucus lysozyme, serum antiprotease, superoxide dismutase, serum bactericidal activity, alternative complement activity, transferrin and Ig in body or tissue fluids [10, 39, 43], which contribute to the elimination of pathogens. Serum proteomic study after *Bacillus* sp. JB-1 supplementation increased transferrin protein quantity in rainbow trout [44]. In human, oral administration of probiotics interacts with intestinal epithelial cells to enhance the production of macrophage chemoattractant protein-1, to send signals to other immune cells characterised by an increment of IgA^+^ cells in the intestine, bronchus, and mammary glands [45]. *Lactobacillus acidophilus* La1 can persist in the gastrointestinal tract and act as an adjuvant to increase serum IgA titre to *Salmonella typhi* Ty21 in human [46]. *Lactococcus lactis* increased the expression of complement receptors and adjuvant potential of *Lactobacillus* GG to elevate the humoral immunity [47]. Lysozyme, defensins, cathelicidins, and phospholipase are antimicrobial peptides secreted by Paneth cells located in the bottom of intestinal crypts [48]. A transmission electron microscopic study depicted *Lactobacillus casei* CRL 431 and *Lactobacillus paracasei* CNCM I-1518 increased Paneth cells activity, apparently increasing the secretion of antimicrobial peptides in intestines [49].

## Probiotics Effects on Cultured Finfish

### Tilapia

Tilapia alone contributed 10% (5.377 million tonnes) of global aquaculture production in 2016 [1]. Nevertheless, its intensive production is more susceptible to stress-related disease and mass mortalities [50], and probiotics are viewed a promising potential solution [51].

The effects of single probiotic *L. plantarum* and its strains CGFM639, CCFM8661, and CR1T5 on tilapia were studied [52-55] (Table 1). Supplementation with 10^7^ to 10^8^ CFU g^–1^ of *L. plantarum* resulted in improvement of growth, feed intake and haemato-biochemical characters, similarly to its other strains CGFM639, CCFM8661 and CR1T5. Among these *L. plantarum* and CR1T5 increased protection against infection, and CGFM639 and CCFM8661 reduced aluminium toxicity and lead accumulation in liver, kidney, spleen, gill, and gonad. A combination of *L. plantarum* N11 + *Bacillus velezensis* H3.1 positively modulated tilapia cellular and humoral immunity [36].

Dietary *Clostridium butyricum* improved final body weight and feed digestion at 1.5 × 10^8^ CFU g^–1^ [56], but not at 10^5^ CFU g^–1^ [57] and protection against *A. hydrophila* and *S. agalactiae*, respectively. This probiotic decreased serum malondialdehyde and diamine oxidase but upregulated immune genes expression levels. Similar parameters were modulated along with increment of immunity, and digestive enzymes were also induced by *B. subtilis* HAINUP40 [58] and *B. licheniformis* DAHB1 [59]. Protection against *S. agalactiae* and *A. hydrophila* was also reported after feeding with a combination of *B. subtilis* and *B. licheniformis* [60] and various strains of *Bacillus* spp. [61], respectively.

Immunity modulation was also confirmed by *B. cereus* as a water supplement at 10^4^ CFU ml−1 [62]. Combination of *Bacillus* with *Pediococcus* sp., *Enterococcus* sp. and *Lactobacillus* sp. did not produce any notable enhancement of digestive enzymes or other serum biochemistry parameters [63]. Expression of inflammatory, antimicrobial and T cell response genes after administration of *B. subtilis* ABP1 is an indication of its oral vaccine property [64]. *Paenibacillus ehimensis* NPUST1 [65] and *Aspergillus oryzae* [66] improved growth and immunological competence, but lowered the levels of glucose and cortisol in blood. Similarly, gut probiotic *Rummeliibacillus stabekisii* modulated the aforementioned parameters with enriched gut microbiota and inhibit *A. hydrophila* and *S. iniae* [67]. In addition, supplementation with *Psychrobacter maritimus* S and *P. namhaensis* S089 showed similar modulation except lower HSP70 [16, 68] and *B. amyloliquefaciens* increased fat digestibility [69]. *B. velezensis* TPS3N, *B. subtilis* TPS4 and *B. amyloliquefaciens* TPS17 [70]; *Bacillus* sp. KUAQ1 and KUAQ2 [71] improved tilapia innate immunity. In addition, intestinal lipase activity and morphometric alteration, viz. micro-villus height and width, goblet cells count, and muscle thickness were reported by the former *Bacillus* probiotics. Administration of *B. subtilis* WB60 with *L. lactis* for 8 weeks resulted in better growth, immunity and immune genes expression, villus height, without changing the haematology [72]. Similar modulations were observed after supplementation with *L. plantarum* L-137 [73] and only gene transcription by *L. plantarum* [74]. The *Bacillus* and *Lactobacillus* probiotics specified above conferred elevated protection against different infectious diseases caused by *E. faecalis*, *A. hydrophila*, and *S. agalactiae*.

Applications of three commercial probiotics, Protexin (multi-strain, 6 × 10^7^ CFU g^–1^), Biogen-S (*B. subtilis*, 10^11^ CFU g^–1^) and Diamond V (*Saccharomyces cerevisiae*, 2.6 × 10^10^ CFU g^–1^) increased growth and feed utilizations, immunity and immune genes transcription at 4^th^ and 8^th^ week [75]. Moreover, commercial multi-probiotics AquaStar Pond and EM resulted in higher immune gene transcription relative to single commercial preparation, MicroPan [76]. Available studies indicated that the commercial probiotics containing single bacterial species, namely AlCare (*B. licheniformis*), Biogen (*B. subtilis*), and BioSaf (*S. cerevisiae*) resulted in improved growth [77] without affecting measured dietary digestion parameters [77, 78] (Table 1). Moreover, the use of commercial probiotics as water supplements enhanced protection against *A. hydrophila* [79].

### Atlantic Salmon

Comparatively less information is available on the effects of probiotics in Atlantic salmon. Available information can be categorized into in vivo and in vitro studies as summarized in Table 2.

Wang *et al*. [80] assessed the effect of *B. velezensis* V4 CGMCC 10149 and *Rhodotorula mucilaginosa* CGMCC 1013 on juvenile salmon raised in a recirculating aquaculture system. After 62 days feeding with different doses of these strains, fish weight gain, immunity as well as disease resistance against *A. salmonicida* improved significantly. In the same species, administration with *P. acidilactici* MA18/5M containing commercial probiotic (Bactocell) upregulated TLR3, TNF-α, and IL-1β, enriched gut microbial community and probiotic count in the intestine [81]. Meanwhile, fish growth rate and protection against infectious pancreatic necrosis virus were not affected by this commercial probiotic. In another study, Bactocell reduced intestinal inflammation, maintaining intestinal homeostasis of Atlantic salmon after inducing inflammation with oxazolone [82].

Trout intestinal *Lactobacillus* (RII and RIII) was administered at a dose of ~10^8^ CFU g^–1^, where RIII enhanced species richness and phylogenetic bacterial community diversity in distal intestine [83]. Moreover, *Lab. fermentum* and *Lab. plantarum* modulated gill epithelium through upraising the number of mucous cells, surface area and lamina propria [84]. *Lactobacillus* was reported to be the dominant genus in the intestine of fish fed probiotics (Table 2). In vitro incubation of salmon foregut with 1 ml *Lab. delbrueckii* subsp. lactis (1.6 × 10^5^ CFU ml^–1^) exhibited that probionts adhere to the proximal intestinal mucus and did not cause any damage to morphology and integrity of the intestine [85]. Pre-incubation with the same probiotic offered protection against *A. salmonicida* infection. Similarly, Kristiansen *et al*. [86] showed that *Carnobacterium divergens* adhered to the proximal intestine following in vitro exposure at a concentration of 10^8^ CFU ml^–1^ for 1 h and inhibited the growth of pathogenic *Y. ruckeri*. In another study, in vitro incubation of *C. divergens* prevented *A. salmonicida*- and *V. anguillarum*-induced damage in the foregut of Atlantic salmon to some extent [87].

### Rainbow Trout

Rainbow trout contributed 2% of total global finfish production in 2016 [1]. Numerous benefits of probiotics have been reported in rainbow trout aquaculture, for example, both *L. plantarum* and its strain KC426951 promoted growth and immunity with some exceptions in heamato-biochemical parameters [88, 89] (Table 3). The former probiotic increased alkaline phosphatase (ALP), IgM, and hemoglobin to a significant level, whereas the latter one only increased white blood cells with no alteration of hemoglobin, mean corpuscular volume, and red blood cells concentration. In contrast to the results reported by Soltani *et al*. [89], some haematological parameters like ALP, triglyceride and total cholesterol depicted improved modulation after *Lab. rhamnosus* treatment [90].

Dietary supplementation with LABs *L. buchneri*, *L. fermentum*, and the yeast *S. cerevisiae* together for 130 days improved innate immunity and immune genes transcription without alteration of heamato-biochemical parameters [91]. Modulation of these parameters was also observed after feeding with various strains of LAB [92, 93]. Administration with *L. plantarum*, *Lac. lactis*, and *Leuconostoc mesenteroides* upregulated IL-8 & 10, TNF-α, and IgT and decreased IL-1β, TNF-α and TLR5 transcription before and after *Lac. garvieae* infection, respectively directly in contrast to results reported by Pérez-Sánchez *et al*. [94] (Table 3).

Combination of *Bacillus* sp. with *Lactobacillus* sp. reportedly elevated humoral immunity, intestinal structure and decreased plasma phenoloxidase [95]. The dietary administration of *Bacillus* sp. and *B. licheniformis* individually or in combination showed improved [96], unchanged [63], and decreased [97] results in weight gain and specific growth rate. Humoral immunity was elevated with *B. subtilis* + *B. licheniformis*, but this combination had no influence on serum biochemical parameters apart from total leukocytes.

Trout gut probiotics *Kocuria* SM1 and *Rhodococcus* SM2 failed to induce changes in growth, digestive enzymes, and haematology, with the exceptions of lowered trypsin and increased hemoglobin levels [30]. In another study, single administration of *Kocuria* SM1 improved immunity and serum biochemistry [98]. Besides these, *Kocuria* SM1-fed rainbow trout fingerlings decreased mortality by 10-28% from 73-92% in response to *V. anguillarum* challenge. Dietary administration of *Enterobacter cloacae* and *B. mojavensis* showed improved protection from *Y. ruckeri*, where survival rate increased to 99.2% compared to 35% in the control group [99]. Probiotics like *E. casseliflavus* positively modulated serum biochemistry by decreasing malondialdehyde and lymphocytes without changing alanine and aspirate aminotransferase [100]. Supplementation of *Bifidobacterium animalis* PTCC-1631 and *Bif. lactis* PTCC-1736 at the lowest dose (10^7^ CFU g^–1^) improved fish body weight and digestibility compared to higher doses and to the control group [101] (Table 3).

Treatment with a commercial probiotic, Proviotic containing *L. bulgaricus* and Aqualase (mixture of *S. cerevisiae* and *S. elipsoedas*) demonstrated better growth and diet digestion [102, 103]. Similarly, PrimaLac composed of *L. acidophilus*, *L. casei*, *E. faecium* and *Bif. bifidium* elevated these two parameters along with protease, amylase and lipase activity in the intestine [15].

### Common Carp

Common carp is among the most important commercially cultured fish in the world contributing 8% of total global finfish aquaculture production in 2016 [1]. Probiotics reportedly affect growth, immunological parameters, and disease protection in common carp [7]. Administration of *L. delbrueckii* for 8 weeks showed improved production, feed intake and various digestive enzymes including protease, amylase, lipase, Na+/K+-ATPase, creatine kinase and γGT activity [104, 105]. Moreover, similar to *L. delbrueckii*, *Lactobacillus* sp. and *Bacillus* spp. modulated immune-gene transcription when used as water supplements [106]. Comparable modulation of parameters was noted after 60 days administration of *L. plantarum* 44^a^, although the probiotic had no effects on lipase and haematological parameters [107]. In another study, same probiotic supplementation unveiled better serum biochemistry, phagocytic and λ-globulin activity in head kidney and protection against *A. hydrophila*, whereas phagocytic activity in spleen remains unchanged [108] (Table 4).

Supplementation with *B. coagulans*, *B. licheniformis* and *P. polymyxa* (MTCC 122) at 10^9^ CFU g^–1^ influenced growth and feed utilizations, innate immunity [109], survival percentage, protection from *A. hydrophila* and *V. harveyi* [110]. Combined applications of *Lactobacillus* sp. with *Bacillus* sp. at 0.75, 1.5 and 2.25 g kg^–1^ improved protease activity in the intestine and modulated water quality parameters like pH, temperature and lowered dissolved oxygen concentration [111]. Administration of *P. acidilactici* [112] and *P. pentosaceus* [113] for 60 and 45 days, respectively, exhibited contrasting results. *P. acidilactici* improved feed conversion ratio, but decreased final body weight and daily weight gain, protease activity and immune gene expression. On the other hand, *P. pentosaceus* improved growth and digestive enzymes activity. However, both probiotics increased innate immunity and serum biochemistry (Table 4). Commercial probiotic mixture viz. Primalac^®^ elicited a similar pattern of results, but only level of white blood cells and serum total protein increased among all haemato-biochemical parameters [114, 115].

### Japanese/Olive Flounder

Olive flounder is among increasingly commercially important cultured marine species in Northeast Asian countries like China, Japan, Korean peninsula and some other countries [11]. This fish is widely accepted all over the world due to fast growth, taste, palatability, wide range of environmental adaptation and disease resistance. In 2018, its production was 37,239 metric ton equaling ~52% of total fisheries production in the Republic of Korea [116].

Extensive studies have focused on the use of single *L. lactis* BFE920 (LLBF) [17, 117], LLBF and *L. plantarum* FGL0001 (LPFG) [17] as well as single and combination of LLBF and LPFG [35] in olive flounder. LLBF and LPFG were isolated from bean sprouts and the hindgut of olive flounder respectively, and their probiotic properties were confirmed in vitro before dietary supplementation. LLBF was also administered orally as vaccine after cloning with *E. tarda* membrane protein and *S. iniae* antigen termed as OmpA, FlgD, and OmpA-FlgD [118] and SiMA [117] respectively (Table 5). These oral vaccines increased transcription levels of cluster of differentiation (CD) 4-1, CD4-2, CD8-α, T-bet, INF-γ, weight (20-21%) and feed digestion. Both vaccines also elevated antigen specific antibodies in fish tissues, as well as protection against *E. tarda* (5 × 10^5^ CFU ml^–1^; LD_80_) and *S. iniae* (10^5^ CFU ml^–1^) producing 82% higher rates of survival relative to controls. Individual supplementation of probiotics LLBF and LPFG at 10^7^ CFU g^–1^ increased expression levels of regulatory and pro-inflammatory genes, respectively [17](Table 5). In addition, gut permeability was increased by LPFG compared to control and LLBF, and immune tone was stable for 30 days. Similar concentration level with combination of LLBF and LPFG increased levels of immunity and immune genes transcription and conferred higher protection against *S. iniae* challenge [35].

Except Sporolac, Lactobacil and mixture of probiotics improved immune parameters and resistance against lymphocystis. In other studies, infectious *S. parauberis*-injected flounder fed mixed probiotics [119] and *Uronema marinum* infected group fed individual *L. plantarum*, *L. acidophilus*, *L. sakei*, *B. subtilis* and *S. cerevisiae* [120] at 0.1% and 2.42 × 10^8^ CFU g^–1^ respectively. In both cases, fish production, haematology and immunity levels were higher in disease infected probiotics treated groups as compared to controls. Among individual probiotics only *L. plantarum* elevated survival in *U. marinum* infected fish compared to other 4 probiotics and control. Another commercial LAB probiotic, *L. fermentum* was supplemented at 0.5% for 8 weeks to compare the effects with yacon, ginger and blueberry at 0.1% [121]. Commercial probiotics showed no effects except higher protection against *S. iniae* infection.

A probiotic *Bacillus* SJ-10 (BSJ-10) was isolated from Korean traditional fermented food [122] and complete genome sequence demonstrated similarity with two well-known olive flounder probiotics, *B. subtilis* and *B. licheniformis* [123, 124]. Dietary supplementation with this probiotic both live [21, 116] and heat-killed (HK)[125] forms at 10^8^ CFU g^–1^ for 8 weeks increased rates of diet digestion and growth. Live and HK BSJ-10 and mixture with *L. plantarum* KCCM 11322 (3:1) protect olive flounder against streptococcosis [21, 43, 125] and edwardsiellosis [116]. Two different forms of BSJ-10 demonstrated no effect on heamto-biochemistry and intestinal structure, but both increased immunity and cytokines production in different localised organs like liver, kidney, gill and spleen (Table 5). Live BSJ-10 increased probiotics count in the intestine, but had no effect on IL-6, CD4-1 and CD4-2 transcription, and were able to ferment β-glucooligosaccharides and barley β-glucan as a synbiotic disease biocontrol model in olive flounder. Treatment with 3:1 *Bacillus* sp. SJ-10 and *L. plantarum* KCCM 11322 at 0.75 + 0.25 × 10^8^ CFU g^–1^ feed resulted in improved growth, immunity and disease resistance in olive flounder [43]. Similarly, feeding with *B. subtilis*, *B. pumilus* and *B. licheniformis* demonstrated identical patterns of response in olive flounder [22]. Among these probiotics, *B. subtilis* and *B. licheniformis* improved disease protection compared to control. In addition, only *B. subtilis* increased fish survival and decreased ammonia concentration (5 days experiment) in controlled culture environments. Jang *et al*. [29] used vegetative cells of BSJ-10 and noted improved growth and feed utilization, and spore-modulated immune genes transcription, leading to improved richness and diversity of intestinal bacterial population.

A study with individual (BSJ-10 and *L. plantarum*) [28] and multi-strain probiotics [126] as supplemented with 30% reduced fish meal diet was found to maintain intestinal homeostasis and immune competence in olive flounder. Both BSJ-10 and *L. plantarum* increased lipase and trypsin, but amylase activity was only improved by BSJ-10. Individual and multi-probiotics also improved concentrations of proteobacteria, actinobacteria, and acidobacteria, and *Lactobacillus*, *Marinilactibacillus* and *Globicatella* in the intestine, while also elevating cytokines transcription levels. Moreover, multiple and individual probiotics had no effects on production and intestinal structure respectively but improved infectious disease resistance of experimentally-cultured fish.

### Grass Carp

Grass carp is predominant among cultured finfishes, comprising 11% of total global aquaculture production in 2016 [1] with production increasing to over 5.7 million tons in 2018; FAO ranked grass carp as the #1 aquacultured species worldwide in 2020 [127]. Several studies have examined the effects of probiotics in grass carp. Dietary *B. licheniformis* FA6 enhanced production, humoral immunity, upregulated immune gene transcription, intestinal morphology and *Aeromonas* infection protection in grass carp [128]. Identical results [129] along with modulation of intestinal microbiome [130] were noted after dietary supplementation of *B. subtilis* Ch9, and both *B. methylotrophicus* WM-1 [131] and *Streptomyces amritsarensis* N1-32 [132] contributed in the modulation of immunity and aeromonosis protection (Table 6).

Individual and mixture of *Shewanella xiamenensis* A-1, *S. xiamenensis* A-2, and *A. veronii* A-7 at 10^8^ CFU g^–1^ feed enhanced cellular and humoral immunity, serum biochemistry, gene transcription and offered *A. hydrophila* protection [133]. Administration of *S. xiamenensis* A-1, *A. veronii* A-7 and *B. subtilis* modulate the gut microbiota either singly or in combination, in which proportions of *Cetobacterium*, *Citrobacter*, *Vibrio*, *Enterococcus* and *Streptococcus* were increased, although a decreasing number was noted for *Pseudomonas* and *Flavobacterium* [134].

Several studies were carried out to examine the effects of probiotics on water quality parameters and microbiota in the culture environment of grass carp. The addition of *P. stutzeri* F11 [135] and *B. licheniformis* BSK-4 [136] in water led to reduction of ammonia, nitrite, total nitrogen and altered microbial populations at 9 and 18 days, respectively (Table 6). In another study, application of individual and mixture of *P. stutzeri* SC221-M and *B. cereus* BSC24 in culture water improved the rearing water quality as revealed by lower levels of total dissolved solids, chemical oxygen demand, ammonium, nitrite and total nitrogen compared to the control after 6-days post-treatment [137]. Moreover, these probiotics increased proteobacteria and decreased bacteroidetes and actinobacteria in ambient water.

### Rohu Carp

*Labeo rohita*, commonly known as Indian major carp or rohu, is among the most cultured fish species in South Asia due to its commercial value, fast growth rate, consumer preferences and comparative simplicity in weaning to artificial diets [138, 139]. Global production of rohu was 1.843 million tons in 2016, contributing 3% of total global finfish production [1]. Weakened immune system and lack of necessary digestive enzymes in juveniles can lead to higher rates of pathogenic bacterial infection and relatively poor feed utilization [138, 140]. Efforts have been made continuously to subdue these problems, including many involving applications of probiotics.

Higher serum IgM levels were recorded after 30 days, and the level decreased after 60 days. Similarly, mixtures of these probiotics with *B. subtilis* VSG1 modulated and generally improved similar immunological parameters, including disease resistance [141] (Table 7). Feeding of *B. subtilis*, *L. lactis* and *S. cerevisiae* mixture at different culture temperatures (28, 31, 34, and 37°C) reduced the degree of apoptosis, augmented the transcription of HSP70, positively modulated haemato-biochemistry and innate immunity compared to the control [142]. Supplementation with *B. methylotrophicus*, *B. amyloliquefaciens* and *B. licheniformis* either singly or in combination elevated digestive enzymes activity, immunity and offered a significant degree of infection protection [143]. Similarly, disease protection and immunity alteration were observed after administration with *B. aerophilus* KADR3 [20], *B. amyloliquefaciens* CCF7 [144] and COFCAU_P1 [145] (Table 7).

Improved protease, amylase and cellulose resulting on good growth in rohu fingerlings were observed after adding *Geothichum candidum* QAUGC01 to rearing water [140]. Similarly, *G. candidum* QAUGC01, *B. cereus* and *G. candidum* QAUGC01 + *B. cereus* at 10^9^ CFU g^–1^ elevated gut microbiome, digestive enzymes and survivability against *A. hydrophila* infection [138]. Similar parameters modulation with improvements in the growth increment were observed after administration of single *E. faecium* QAUEF01 and its combination with *G. candidum* QAUGC01 [146] and *S. cerevisiae* [147].

## Probiotics Application under Farming Condition

Most studies on probiotics were conducted under laboratory conditions, in which water and ambient environmental parameters are subject to rigid control. The role of probiotics in open systems under farming conditions remains minimally studied. While performance of probiotics under uncontrolled field levels is subject to uncertainty, commercial manufacturers of probiotics frequently boast that their products are highly effective for farm level application.

Tilapia cultured in earthen ponds treated with two doses of *B. pumilus* and a commercial probiotic for 8 months showed increase growth, immunity and haematological parameters, and *A. hydrophila* infection protection [79](Table 1). Artificially cultured fishes are subjected in the hatchery to water quality and other parameters that can compromise the consistent production of healthy juveniles [148], although probiotic applications among broodstock, embryos and larvae have been studied inadequately. Female rainbow trout brood stock in raceway ponds supplemented with multi-probiotics before spawning season demonstrated production of eggs with higher diameters, increased fecundity, fertilization and hatching, yolk sac absorption, and eye development [14]. These results are consistent with beneficial responses to multiple-species LAB probiotic applications in the zebrafish hatchery, which led to consistent improvements in every measurable parameter of larval performance [149]. Moreover, multi probiotics supplementation in rainbow trout during grow out in floating cages led to improved haemato-immunological responses and operational taxonomic units in the intestine [150] (Table 3).

Common carp (2.75 g) cultured in raceways and fed with *B. sublitis* for 180 days at 4 × 10^6 & 8^ CFU g^–1^ showed elevated production and feed utilizations as contrasted with controls [151]. Moreover, probiotics increased muscle RNA-DNA ratio along with crude lipid, protein, and ash content in the body (Table 4). Single supplementation of *L. lactis* BFE920 at different concentrations increased protection against streptococcosis (68-77%) after 2 weeks. Subsequently, 2.5 × 10^7^ CFU g^–1^ was chosen for field experiment for 3 months in 12,000 olive flounder [152]. That experiment revealed that probiotics fed group displayed improved immunity and immune related gene expression, resulting in 65.7% survival relative to 5.7% in control group when challenged with *S. iniae* (Table 5).

Cage culture of grass carp (~44 g) for 56 days with *B. natto* NT supplementation improved body weight and feed utilization [153]. Myostatin and myocyte enhancer factor C were downregulated, whereas micro-RNA (miR)-1a, miR-181a, miR-23a, and miR-206 transcription were upregulated compared to control group (Table 6). Rohu carp in earthen ponds displayed improved immunity and digestive enzyme activities along with enhanced tissue HSP70 expression after 11 weeks feeding with encapsulated *G. candidum* QAUGC01 [154]. Multi-probiotics administration in that species in outdoor tanks resulted in higher survival of hatchlings and fry at 8 and 38 days, respectively but those differences were no longer detectable after 68 days [155] (Table 7).

## Research Gaps and Concluding Remarks

Some urgency is attached to the transition from the use and at times excessive use of antibiotics to control pathogens in aquaculture. The effectiveness of antibiotics is counteracted by intense selection favouring resistance, leading to the emergence of dangerously capable pathogens. The hazards of routine antibiotic applications are well-recognized now, and alternatives are critically needed. Probiotics are being marketed to aquaculture professionals, but it is generally true that their effectiveness in any given situation is uncertain.

Probiotics are typically found among microorganisms inhabiting the digestive tracts of healthy aquatic species, and their presence has been found to induce beneficial changes such as improved growth, immunity, and survival. Although the concept of promoting improved health by altering the population structure of the microbiome has gained favour, the mechanisms involved are far from clear. Probiotic organisms decrease the likelihood of the impairment or mortality of culture subjects by pathogens through a poorly understood complex of mechanisms, and more specific understanding of these mechanisms is highly desired. Interactive mechanisms of probiotic species with intestinal cells of host subjects require further investigation and analysis.

Probiotics contribute to the development of an unfavourable environment for the proliferation of pathogens, thereby disabling opportunistic microbes including many pathogens, thereby reducing the prevalence of outbreaks of infectious organisms. This can happen by direct and indirect means, for example by generating metabolites that are inhibitory to pathogens, or that promote activation of the immune system, or sometimes simply by competing for nutrients and environmental niches. As discussed above, activation of growth- and immune-related genes have been observed and may be more symptomatic of beneficial health effects than unknown causative factors. Further study of the mechanisms resulting in beneficial impacts of probiotic organisms may lead to more effective treatments and ultimately to improved environmental management of commercially cultured fishes and invertebrates.

## Figures and Tables

**Table 1:** Effects of probiotics supplementation on growth, feed utilizations, immunological and haemato-biochemical parameters, immune related gene expression and disease resistance in tilapia (Oreochromis niloticus).

Probiotics	Mode of administration and dosage	Duration	Effects on O. niloticus	References
*Lactobacillus plantarum*	Dietary supplementation at 10^7^ CFU g^–1^	60 days	GP _FBW, WG, & SGR_↑; FUP _FCR_↓; IP _TAC, RB, & SL_↑, _and NO & MDA_ ↓; HBP _Ht, TSP, Alb, Glb, & Glu_↑; IDR _*Aeromonas sobria*_↑	[52]
*L. plantarum* CGFM639	Dietary supplementation at 10^8^ CFU g^–1^	4 weeks	GP _WG_↑; FUP _FCR_↓; IP _SOD, GPx, CAT, & TAC_↑, _and MDA_↓; HBP _RBC & WBC_↑, _and ALT_↓; Aluminum toxicity↓	[53]
*L. plantarum* CCFM8661	Dietary supplementation at 10^8^ CFU g^–1^	4 weeks	GP _FBW, WG, & SGR_↑; FUP _FCR_↓; IP _MPO, SL, GPx, & TAC_↑, _and MDA_↓; DEA _AmA & PrA_↑; PbAT _Kidney, Liver, Spleen, Gill, Brain, Gonad, & Muscle_↓	[54]
*L. plantarum* CR1T5	Dietary supplementation at 10^8^ CFU g^–1^	2 months	GP _FBW, WG, & SGR_↑; FUP _FCR_↓; IP _SL, ACA, PcA, & RB_↑; IDR _*Streptococcus agalactiae*_ ↑	[55]
*L. plantarum* N11 & *Bacillus velezensis* H3.1	Dietary supplementation at 10^7^ & 10^8^ CFU g^–1^	30 days	GP _WG, FBW, & SGR_↑; FUP _FCR_↓; IP_ACA, PcA, RB, SL, & SMLA_↑; IDR _*S. agalactiae*_↑	[36]
*Clostridium butyricum*	Dietary supplementation at 1.5×10^8^ CFU g^–1^	8 weeks	GP _FBW, WG, & SGR_↑; FUP _ADC_↑, and _FCR_↓; IP _SOD, SL, & CAT_↑; HBP _TSP, Ht, Hb, AST, ALT, Lymphocyte, & Monocyte_↑; mVH↑; SR→; IDR _*A. hydrophila*_↑	[56]
*C. butyricum*	Dietary supplementation at 10^4^ to 10^7^ CFU g^–1^	56 days	GP _FBW & SGR_↑; FUP _FCR_→; IP _TAC_↑, _and MDA & DAO_↓; IRGE _IL-8, TNF-α, MyD88, TLR2, & IRAK-4_↑, and _IL-10_→; IDR _*S. agalactiae*_↑	[57]
*B. subtilis* HAINUP40	Dietary supplementation at 10^8^ CFU g^–1^	8 weeks	GP _FBW, WG, & SGR_↑; FUP _FCR_↓; IP _RB, TAC, & SOD_↑; DEA _PrA & AmA_↑; IDR _*S. agalactiae*_↑	[58]
*B. licheniformis* DAHB1	Dietary supplementation at 105 & 10^7^ CFU g^–1^	4 weeks	GP _WG & SGR_↑; FUP _FCR_↓; HBP _TSP & ALP_↑; IP _SL, RB, MPO, GPx, & SOD_↑; IDR _*A. hydrophila*_ ↑	[59]
*B. cereus*	Water & dietary supplementation at 10^4^ & 10^8^ CFU g^–1^, respectively	42 days	IP _SL, MPO, & SOD_↑; HBP _ALP_↑; GutM↑	[62]
*B. subtilis* + *B. licheniformis*	Dietary supplementation at 3 to 7 g Kg^–1^ feed	4 weeks	GP _WG & SGR_↑; FUP _FCR_↓; IP _SL, SOD, & CAT_↑; IRGE _β-defensin, TGF-β, & HSP70_↑; DEA _PrA_↓; IDR _*S. agalactiae*_↑	[60]
*B. amyloliquefaciens*	Dietary supplementation at 60 mg/kg feed	42 days	GP _WG & SGR_↑; FUP _FCR_↓; Fat digestibility↑	[69]
*Bacillus* sp., *Pediococcus* sp., *Enterococcus* sp. & *Lactobacillus* sp.	Dietary supplementation 10^6^ & 2.3×10^6^ CFU g^–1^	8 weeks	GP _FBW, WG, & SGR_↑; FUP _FCR & PER_↑, _and ADC_→; IP _RB→, and GPx_↓, _and ACA & CAT_↑; HBP _Ht, WBC, & RBC_→; DEA _AmA, Lipase, Trypsin, & Chymotrypsin_→; mVH↑	[63]
*Paenibacillus ehimensis* NPUST1	Dietary supplementation at 10^6^ & 10^7^ CFU g^–1^	2 months	GP _WG_↑; FUP _FCR & FER_ ↑; IP _PcA, SOD, & RB_↑; IRGE _IL-1β & TNF-α_↑; SR↑; IDR _*A. hydrophila*_ & _*S. iniae*_↑	[65]
*Aspergillus oryzae*	Dietary supplementation at 10^6^ & 10^8^ CFU g^–1^	60 days	GP _FBW, WG, & SGR_↑; FUP _FER_↑; IP _RB, IgM, SL, SBA, MDA, SOD, GPx & PcA_↑; HBP _TSP_↑, _and Glu & Cortisol_↓; IRGE _IL-1β, TNF-α, & HSP70_↑; SR→	[31]
*Rummeliibacillus stabekisii*	Dietary supplementation at 10^6^ & 10^7^ CFU g^–1^	8 weeks	GP _WG & FBW_↑; FUP _FCR_↓, _and FER_↑; IP _PcA, RB, SL, & SOD_↑; IRGE _IL-1β, TNF-α, TGF-β, & HSP70_↑; SR↑; DEA _PrA, Xylanase, & Cellulase_↑; GutM _Bacillus & Lactobacillus_↑, _and *Staphylococcus* & *Streptococcus*_↓; IDR _*A. hydrophila*_ & _*S. iniae*_↑	[67]
*Psychrobacter namhaensis* S089	Dietary supplementation at (2.8 & 5.6) ×10^7^ CFU g^–1^	50 days	GP _FBW, WG, & SGR_↑; FUP _FCR_↓, _and PER, & PPV_↑; IP _RB ↑; HBP TSP, RBC, WBC, Hb, Ht, Alb, Glb, AST, ALT, & LDH_↑; IRGE _IL-4 & IL-12_↑; DEA _PrA, AmA & Lipase_↑	[68]
*Psychrobacter maritimus* S	Dietary supplementation at (3.3 & 6.6) ×10^8^ CFU g^–1^	50 days	GP _FBW, WG, & SGR_↑; FUP _FCR_↓, _and PER_↑; IP _PcA, ACA, SL, & SOD_↑; HBP _RBC, TSP, Alb, Glb, Hb, & Ht_↑, _and ALT, & AST_↓; IRGE _IL-4 & IL-12_↑, _and HSP70_↓; DEA _PrA, AmA, & Lipase_↑	[16]
*B. velezensis* TPS3N; *B. subtilis* TPS4 & *B. amyloliquefaciens* TPS17	Dietary supplementation at 10^8^ CFU ml^–1^	4 weeks	IP _NO, SL, IgM, CAT, & SOD_↑; HBP _ALP_↑; DEA _AmA→, and Lipase_↑; mVH↑; IDR _*A. hydrophila*_ ↑	[70]
*Bacillus* sp. KUAQ1 & *Bacillus* sp. KUAQ2	Dietary supplementation at (1, 3, & 5) ×10^8^ CFU g^–1^	8 weeks	GP _WG_↑, _and SGR_↓; FUP _FCR_↓; IP _PcA, SOD, & ACA_↑; IDR _*S. agalactiae*_↑	[71]
*B. subtilis* WB60 & *Lactococcus lactis*	Dietary supplementation at 10^7^ & 10^8^ CFU g^–1^	8 weeks	GP _ & SGRWG_↑; FUP _FER & PER_↑; HBP _AST, ALT, TSP, & Glu_→; IP _SL, SOD, & MPO_↑; IRGE _INF-γ, IL-1β, HSP70, & TNF-α_↑; mVH↑; IDR _*A. hydrophila*_↑	[72]
*Bacillus* spp. (ANSCI9, BFAR9, RM3, and RM10)	Dietary supplementation at 10^8^ CFU g^–1^	30 days	GP _SGR, RGR_↑; FUP _FCR_↓; IDR _*A. hydrophila*_ ↑; SR ↑	[61]
*L. plantarum* L-137	Dietary supplementation at 2×10^11^ CFU g^–1^ & 50mg Kg^–1^	30 days	HBP _TSP, Alb, Glb, Hb, RBC, & WBC_↑, _and AST, ALT, ALP, Creatinine, Urea, & Bilirubin_↓; IP _CAT, GPx, SL, & PcA_↑; IRGE _INF-γ_→, _and IL-8 & IL-1β_↑, _and CASP3 & HSP70_↓	[66]
*L. plantarum*	Dietary supplementation at 1.09×10^9^ CFU Kg^–1^	56 days	GP _WG_→; IRGE _IL-10, IL-17F, IL-8, & IL-1_β↑; GutM↑; IDR _*Enterococcus faecalis*_↑	[74]
Protexin (*L. plantarum*, *L. rhamnosus*, *Bifidobacterium bifidum*, *E. faecium*, *Candida pintolepesii*, & *Aspergillus oryzae*), Biogen-S (*B. subtilis*), Diamond V (*Saccharomyces cerevisiae*)	Dietary supplementation at 6×10^7^, 10^11^ & 2.6×10^10^ CFU g^–1^	8 weeks	GP_FBW, WG, & SGR_↑; FUP _FCR_↓; IP _RB, & SL_↑; HBP _TSP, Alb, Glb, A/G Ratio, Hb, Ht, RBC, & WBC_↑, _and Glu, ALT, & AST_↓; IRGE _IL-1β & TNF-α_↑; IDR _*A. hydrophila*_↑	[75]
AlCare (*B. licheniformis*)	Dietary supplementation at 2×10^7^ to 4.4×10^6^ CFU g^–1^	10 weeks	GP _FBW, WG, & SGR_↑; FUP _FCR_→; IP _SL, SOD, & ACA_↑; SR→; IDR _*S. iniae*_ ↑	[77]
DVAQUA (*S. cerevisiae*)	Dietary supplementation at 2.0g Kg^–1^ feed	8 weeks	GP _WG & SGR_→; FUP _FCR_→; IP _SL, RB, PcA, & ACA_↑; GutM↑; SR→	[78]
Organic GreenTM & *B. pumilus*	Water supplementation at 10^8^ CFU ml^–1^	1, 2, & 8 months	GP _WG_↑; HBP _Ht, TLC, DLC, Neutrophils, Lymphocytes, Monocytes, Eosinophils, & Basophils_ →; IP _RB & RLP_↑; SR↑; IDR _*A. hydrophila*_↑	[79]
AquaStar Pond (*Bacillus* sp., *Pediococcus* Sp., *Enterococcus* Sp.); EM (*Rhodopseudomonas* spp., *Lactobacillus* spp., *Saccharomyces* spp.); MicroPan (*Bacillus* spp.)	Dietary supplementation at 0.0015 g m^− 3^ day^− 1^; 10 ml m^− 3^ day^− 1^; 0.002 g m^− 3^ day^− 1^	60 days	GP _FBW, WG & SGR↑_; FUP _FER & PER_↑; IRGE _GHR, IL-1β, TNF-α & IGF-1_↑, HBP _Alb, Glb, Glu, Hb, WBC, RBC, Lymphocyte, & Monocyte_↑; IP _SL & PcA_↑	[76]

Symbol →, no change; ↑, increase; ↓, decrease versus controls.

A/G: Albumin/Globulin; ACA: Alternative complement activity; ADC: Apparent digestibility coefficient; Alb: Albumin; ALP: Alkaline phosphatase; ALT: Alanine amino transferase; AmA: Amylase activity; AST: Aspartate amino transferase; CASP3: Caspase 3; CAT: Catalase; DAO: Diamine oxidase; DEA: Digestive enzyme activities; FBW: Final body weight; FCR: Feed conversion ratio; FER: Feeding efficiency ratio; FUP: Feed utilization parameters; Glb: Globulin; Glu: Glucose; GP: Growth parameters; GPx: Glutathione peroxidase; GutM: Gut microbiota; Hb: Haemoglobin; HBP: Haemato-biochemical parameters; HSP: Heat shock protein; Ht: Hematocrit; IDR: Infectious disease resistance; IgM: Immunoglobulin M; IL: *Interleukin*; INF: Interferon; IP: Immunological parameters; IRAK: Interleukin 1 receptor associated kinase; IRGE: Immune related gene expression; LDH: Lactate dehydrogenase; MDA: Malondialdehyde; MPO: Myloperoxidase; mVH: micro-Villous height; MyD: Myeloid differentiation factor; NO: Nitric oxide; PbAT: Pb accumulation in tissues; PcA: Phagocytic activity; PER: Protein efficiency ratio; PPV: Protein productive value; PrA: Protease activity; RGR: Relative growth rate; RB: Respiratory burst (NBT assay); RBC: Red blood cell; RLP: Relative level of protection; SBA: Serum bactericidal activity; SGR: Specific growth rate (%); SL: Serum lysozyme; SMLA: Skin mucus lysozyme activity; SOD: Superoxide dismutase; SR: Survival rate; ST: Salinity tolerance; TAC: Total anti-oxidant capacity; TGF: Transforming growth factor; TLC: Total leucocytic count; TLR: Toll-like receptor; TNF: Tumor necrosis factor; TSP: Total serum protein; WBC: White blood cells; WG: Weight gain (%).

**Table 2:** Effects of probiotic supplementation on growth, feed utilizations, immunological and haematobiochemical parameters and disease resistance in Atlantic salmon (*Salmo salar*) aquaculture.

Probiotics	Mode of administration and dosage	Duration	Effects on *S. salar*	References
*Bacillus velezensis* V4 CGMCC 10149 (BVV4) & *Rhodotorula mucilaginosa* CGMCC 1013 (RM)	Dietary supplementation at BVV4 (5 × 10^6^) + RM (5 × 10^7^), BVV4 (1.5 × 10^7^) + RM (1.5 × 10^8^), & BVV4 (2.5 × 10^7^) + RM (2.5 × 10^8^) CFU g^–1^	62 days	GP _FBW, WG, & SGR_↑; FUP _FCR_↓; IP _SOD, GPx, CAT, TAC, ACP, & IgM_↑_, MDA & NO_↓_, and_ _SL_ →; HBP _ALT & AST_↓; IDR _*Aeromonas salmonicida*_↑; SR↑	[80]
Bactocell (*Pediococcus acidilactici* MA18/5M)	Dietary supplementation at 1.19 × 10^6^ CFU g^–1^	12 weeks	GP _FBW & SGR_ →; IRGE _MX-1, TLR3, TNF-α, & IL-1β_↑_,__and HSP70 & PCNA_ →; GutM _Proteobcteria_↑, _and Fusobacteria_↓; PCI↑; IDR _IPNV_ →	[81]
Bactocell (*P. acidilactici* MA18/5M)	Dietary supplementation at 10^10^ CFU Kg^–1^	6 weeks	IRGE _MUL-1β, IL-1β, TNF--α, & INF-γ_↓; Gut Morphology _Goblet cells, intraepithelial lymphocytes (IELs), Supranuclear vacuoles in the villi, & immune cells_↑	[82]
*Lactobacillus* RII & RIII	Dietary supplementation at ∼ 10^8^ CFU g^–1^	20 days	GutM _Firmicutes,_ _Tenericutes, Proteobacteria, Actinobacteria, & Spirochaetes_↑	[83]
*Lab. fermentum* and *Lab. plantarum*	Dietary supplementation at 10^8^ CFU g^–1^	38 days	GME and GNE↑; VH & LPW↑	[84]
*Lab. delbrueckii* subsp. *lactis*	*In vitro* incubation at 1.6 × 10^5^ CFU ml^–1^	30 min	PCI↑; IDR _*A. salmonicida* subsp. *salmonicida*_↑	[85]
*Carnobacterium divergens*	*In vitro* incubation at 10^8^ CFU ml^–1^	1 h	PCI↑; Gut Morphology _Goblet cell_ → Pathogenic antagonism _*Y. ruckeri*_↑	[86]
*C. divergens*	*In vitro* incubation at 6 × 10^4^ & 6 × 10^6^ CFU ml^–1^	1 h	IDR _*A. salmonicida* & *V. anguillarum*_↑	[87]

Symbol →, no change; ↑, increase; ↓, decrease versus controls.

ACP: Acid phosphatase; ALT: Alanine aminotransferase; AST: Aspartate aminotransferase; CAT: Catalase; FBW: Final body weight; FCR: Feed conversion ratio; FUP: Feed utilization parameters; GME and GNE: The area and number of mucous cells/ μm2 gill epithelium; GP: Growth parameters; GPx: Glutathione peroxidase; GutM: Gut microbiota; HBP: Haemato-biochemical parameters; HSP70: Heat shock protein; IDR: Infectious disease resistance; IgM: Immunoglobulin M; IL: *Interleukin*; INF: Interferon; IP: Immunological parameters; IPNV: Infectious pancreatic necrosis virus; IRGE: Immune related gene expression; LPW: Narrower lamina propria; MDA: Malondialdehyde; MUL: Mitochondrial ubiquitin ligase activator of NFκB1; MX-1: Interferon-induced GTP-binding protein; NO: Nitric oxide; PCI: Probiotic count in the intestine; PCNA: Proliferating cell nuclear antigen; SGR: Specific growth rate (%); SL: Serum lysozyme; SOD: Superoxide dismutase; SR: Survival rate; TAC: Total anti-oxidant capacity; TLR: Toll-like receptor; TNF: Tumor necrosis factor; VH: villi height; WG: Weight gain (%).

**Table 3:** Effects of probiotic supplementation on growth, feed utilizations, immunological and haemato-biochemical parameters, immune related gene expression and disease resistance in rainbow trout (*Oncorhynchus mykiss*).

Probiotics	Mode of administration and dosage	Duration	Effects on *O. mykiss*	References
*Lactobacillus plantarum* KC426951	Dietary supplementation at 2×10^7^ CFU g^–1^	72 days	GP _FBW & WG_↑; FUP _FCR_↓; HBP _WBC_↑_, and_ _Hb,_ _MCV, MCH, MCHC, & RBC_→	[88]
*L. plantarum*	Dietary supplementation at 10^8^ CFU g^–1^	60 days	GP _FBW, WG, & SGR_↑; FUP _FCR_↓_,__and PER_↑; IP _IgM & ACA_↑; HBP _RBC, MCH, MCHC, MCV, WBC, Heterophil, & Lymphocytes_→	[89]
*L. rhamnosus*	Dietary supplementation at 10^9^ & 10^11^ CFU g^–1^	30 days	HBP _TC, ALP, TG, TSP, & Ht_↑	[90]
*L. buchneri, L. fermentum* & *Saccharomyces cerevisiae*	Dietary supplementation at 10^7^ CFU g^–1^	130 days	GP _WG & SGR_↑; FUP _FCR_↓; IP _RB_↓; HBP _TSP, Alb, Glb, TC, TG, & RBC_→_,__and_ _WBC_↑; IRGE _TNF-α & IL-1β_↑	[91]
*L. delbrukei* subsp. *bulgaricus*, *L. acidophilus* & *Citrobacter farmeri*	Dietary supplementation at 5×10^7^ CFU g^–1^	60 days	GP _FBW, WG, & SGR_↑; FUP _FCR_↓_,__and FER_↑; IP _SL, SBA, & ACA_↑_,__and RB_↓; HBP _Ht, Hb, ALP, WBC, MCV, & MCH_↑_, and RBC_→_, and MCHC_↓_;_ IRGE _IL-1β, IL-8, IL-10, IGF-1, γ-GTP, & FATP_↑; DEA _Trypsin, AmA, & Lipase_↑_,__and PrA_→	[93]
*L. delbrueckii* subsp. *bulgaricus*	Dietary administration at 10^8^ CFU g^-1^	66 days	GP _WG, DWG & SGR_↑; FUP _FER & PER_↑ _FCR_↓; HBP _Alb, ALP-liver_↓; IP _SOD, CAT, GSH_↑	[92]
*L. plantarum*, *Lactococcus lactis*, & *Leuconostoc mesenteroides*	Dietary administration at 10^6^ CFU g^-1^	36 days	IRGE _IL-10, IL-1β, TNF-α, IgT, & IL-8_↑_,__and IL-1β, TNF-α, & TLR5 (after infection)_ ↓; IDR _*Lac. garvieae*_↑	[94]
*Bacillus* sp. + *Pedicoccus* sp., *Enterococcus* sp. + *Lactobacillus* sp. + *P. acidilactici*	Dietary supplementation at 8.6×10^5^, 1.6×10^6^, 2.6 × 10^4^ & 7.2 ×10^4^ CFU g^–1^	8 weeks	GP _FBW & WG_↑; FUP _FCR_↓_,__and PER_↑; IP _SL & ACA_↑_,__and_ _MPO_↓; mVH↑	[95]
*B. subtilis*, *B. licheniformis*	Dietary supplementation at (7.79, 8.36, 8.05, & 8.23) ×10^1^ CFU g^–1^	10 weeks	GP _WG_↓_,__and SGR_↑; FUP _FCR_↓_, and PER_↑; IP _SL_↑; HBP _Ht, Lymphocytes, Granulocytes & Thrombocytes_→_,__and WBC_↑; TVC↑	[97]
*B. subtilis* & *B. licheniformis*, & *B. subtilis* + *B. licheniformis*	Dietary supplementation at 2×10^9^ CFU kg^–1^	8 weeks	GP _WG & SGR_↑; FUP _FER & PER_↑; IP _SOD, MPO, RB, & SL_↑; HBP _AST, ALT, Glu, & TC_→	[96]
*B. subtilis* & *B. cereus toyoi*	Dietary supplementation at 6×10^3^ & 1.5×10^6^ CFU g^–1^	20 weeks	GP _WG & SGR_→; FUP _FER & PER_→; IP _SL_↓_,__and ACA_↑; mVH→	[63]
*B. subtilis* ABP1 & ABP2	Dietary supplementation 10^5^, 10^6^, and 10^7^ CFU g^–1^	1 week	IP _IgM_↑; HBP _MCH II_↑	[64]
*Kocuria* SM1 & *Rhodococcus* SM2	Dietary supplementation at 10^7^(SM1) or 10^8^ (SM2) CFU g^–1^	14 days	GP _FBW & SGR_→; FUP _FCR & PER_→; DEA _PrA_→_,__& Trypsin_↓; HBP _Hb_↑_,__and_ _ALP, ACP, Urea, Creatinine, & Glu_→	[30]
*Kocuria* SM1	Dietary supplementation at 10^8^ CFU g^–1^	2 weeks	IP _SBA, RB, & SL_↑; HBP _TSP & WBC_↑; IDR _*Vibrio anguillarum*_↑;	[98]
*Enterobacter cloacae* & *B. mojavensis*	Dietary supplementation at 10^8^ CFU g^−1^	60 days	HBP _Hb, WBC, Neutrophils, Lymohocytes, & Monocytes_↑_,__and Eosinophils & RBC_→; IDR _*Y. ruckeri*_↑; SR↑	[99]
*Enterococcus casseliflavus*	Dietary supplementation at 10^7^, 10^8^, 10^9^ CFU g^–1^	8 weeks	GP _FBW, WG, & SGR_↑; FUP _FCR_↓; IP _IgM & RB_↑; HBP _TSP_, _RBC, WBC, Ht, Alb, Hb, MCH, MCHC, Neutrophils, Monocyte, & Lysozyme_↑_, and MCV & Lymphocytes_↓_,__and_ _AST, ALT, LDH, & Glb_→	[100]
*Bifidobacterium animalis* PTCC-1631 & *Bif. lactis* PTCC-1736	Dietary supplementation at (1, 2, & 3) ×10^7^ CFU g^–1^	8 weeks	GP _FBW, WG, & SGR_↑; FUP _FI & FCR_↓ _and FCE_→; GutM↑	[101]
Bio Aqua (*P. acidilactici*, *E. faecium*, *B. subtilis*, *L. acidophilus*, *L. plantarum*, *L. casei*, *L. rhamnosus*, *B. bifidum* & *S. cerevisiae*)	Dietary supplementation at (1, 2, & 4) ×10^9^ CFU g^–1^	8 weeks	GP _FBW, WG, & SGR_↑; FUP _PER_↑_,__and_ _FCR_↓; RP _ED, RF, FR, & HR_↑; HBP _TSP, Alb, Glb, & A/G Ratio_↑, _and RBC, WBC, Hb, Ht, Lymphocyte, Monocyte, Eosinophils, Basophils, MCV, MCH, & MCHC_→_,__and_ _Glu, TG, & TC_↓	[14]
Proviotic (*L. bulgaricus*)	Dietary supplementation 460 mg Kg^–1^	60 days	GP _FBW & SGR_↑; FUP _FCR_↓; HBP _Glu_↑_,__and_ _Urea, TSP, Alb, ALT, ALP, Ca, P, Mg, TG, & TC_↓	[103]
PrimaLac (*L. acidophilus*, *L. casei*, *E. faecium*, & *Bif. bifidium*)	Dietary supplementation 1.5 g Kg^–1^	8 weeks	GP _FBW, WG, & SGR_↑; FUP _FCR_↓; HBP _LDH, ALP, ALT, AST,_ _TG, Glu, Cortisol, & TC_↓; DEA _PrA, AmA, & Lipase_↑; SR↑	[15]
Aqualase (*S. cerevisiae* & *S. elipsoedas*)	Dietary supplementation at 10^10^ CFU g^–1^	8 weeks	GP _WG & SGR_↑; FUP _FCR_↓; IP _SL, IgM, & RB_↑_;_ HBP _TSP_↑_, and ALP_→; DEA _Trypsin, AmA, & Lipase_↑	[102]
*Bacillus* sp., *Pediococcus* sp., *Enterococcus* sp. & *Lactobacillus* sp.	Dietary supplementation 2×10^9^ CFU kg^–1^	9 weeks	GP _FBW & WG,_ →; IP _ACA & GPx_ ↑_,__and CAT & SL_→; GutM→	[150]

Symbol →, no change; ↑, increase; ↓, decrease versus controls.

A/G: Albumin-Globulin; ACA: Alternative complement activity; ACP: Acid phosphatase; ADC: Apparent digestibility coefficient; Alb: Albumin; ALP: Alkaline phosphatase; ALT: Alanine transaminase, AmA: Amylase activity; AST: Aspartate amino transferase; Ca: Calcium; CAT: Catalase; DWG: Daily weight gain; DEA: Digestive enzyme activity; ED: Egg diameter; FATP: Fatty acid transport protein; FBW: Final body weight; FCE: Feed conversion efficiency; FCR: Feed conversion ratio; FER: Feed efficiency ratio; FLs: Fingerlings; FR: Fertilization rate; FUP: Feed utilization parameters; Glb: Globulin; Glu: Glucose; GP: Growth parameters; GSH: Glutathione; GPx: Glutathione peroxidase; GTP: Gamma glutamyl transpeptidase; GutM: Gut micorbiota; Hb: Haemoglobin; HBP: Haemato-biochemical parameters; HR: Hatching rate; Ht: Hematocrit; IDR: Infectious disease resistance; Ig: Immunoglobulin; IGF: Insulin-like growth factor; IL: *Interleukin*; IP: Immunological parameters; IRGE: Immune related gene expression; LDH: Lactate dehydrogenase; LPV: Lipid productive value; MCH: Mean corpuscular haemoglobin; MCHC: Mean corpuscular haemoglobin concentration; MCV: Mean corpuscular volume; Mg: Magnesium; MPO: Myloperoxidase; mVH: micro- Villous height; P: Potassium; PcA: Phagocytic activities; PER: Protein efficiency ratio; PPO: Plasma peroxidase; PPV: Protein productive value; PrA: Protease activity; RB: Respiratory burst (NBT assay); RBC: Red blood cells; RF: Relative fecundity; RP: Reproductive parameters; SAP: Serum antiprotease activity; SBA: Serum bactericidal activity; SGR: Specific growth rate (%); SL: Serum lysozyme; SOD: Superoxide dismutase; SR: Survival rate; TC: Total cholesterol; TG: Triglyceride; TLR: Toll like receptor; TNF: Tumor necrosis factor; TSP: Total serum protein; TVC: Total viable counts; VCCS: Vertebral column compression syndrome; WBC: White blood cell; WG: Weight gain (%).

**Table 4:** Effects of probiotic supplementation on growth, feed utilizations, immunological and haematobiochemical parameters and disease resistance in common carp (*Cyprinus carpio*).

Probiotics	Mode of administration and dosage	Duration	Effects on *C. carpio*	References
*Lactobacillus delbrueckii*	Dietary supplementation at 10^5^ to 10^8^ CFU g^–1^	8 weeks	GP _FBW & WG_↑; FUP _FCR_↓; HBP _ALP_→; IP _IgM, SL,_ _MPO, ACP, SOD, CAT, GPx, & TAC_↑_, and MDA_↓; IRGE _TNF-α, IL-8, IL-1β, & NF-κBp65_↓_,__and IL-10 & TGF-β_↑; IDR _*Aeromonas hydrophila*_↑; SR↑	[105]
*L. delbrueckii*	Dietary supplementation at 10^5^ to 10^8^ CFU g^–1^	8 weeks	GP _FBW, WG, and SGR_↑; FUP _PER_↑; DEA _PrA, AmA, Lipase, Na_^+^_/K_^+^-_ATPase, CK, & γGT_↑	[104]
*Lactobacillus* sp., *Nitrosomonas* sp. and *Bacillus* spp.	Water supplementation at 10^8^ CFU ml^–1^	10 days	IRGE _TNF-α, CD4_^+^_and CD8_^+^↑	[106]
*L. plantarum* 44^a^	Dietary supplementation at (1.5, 3, & 4.5) ×10^6^ CFU mg^−1^	60 days	GP _SGR & TGC_↑; FUP _PER_↑_,__and_ _FCR_ ↓; IP _SL_↑; DEA _PrA & AmA_↑_,__and Lipase_ →; HBP _RBC, Ht, Hb, Leucocyte, MCH, MCHC, & Glb_↑_, and_ _Cortisol & Glu_↓_,__and_ _Alb_ →	[107]
*L. plantarum*	Dietary supplementation at 10^8^ CFU g^−1^	14 days	IP _PcA (Head kidney) & λ-globulin_↑_, and_ _SBA_↓_,__and_ _PcA (Spleen),_ _RB,_ _SL, & CP_→; HBP _B lymphocytes & TSP_↑; IDR _*A. hydrophila*_ ↑	[108]
*Bacillus coagulans* MTCC 9872, *B. licheniformis* MTCC 6824 & *Paenibacillus polymyxa* MTCC 122	Dietary supplementation at 10^9^ CFU g^−1^	80 days	GP _FBW & SGR_↑; FUP _FCR & PER_↑; IP _SL, RB, &_ _MPO_↑; IDR _*A. hydrophila* & *Vibrio harveyi*_↑; SR↑	[110]
*B. coagulans*	Dietary supplementation at (1, 2, & 4) ×10^7^ CFU g^–1^	45 days	GP _FBW & WG_↑; IP _ACA, SL,_ _MPO & RB_↑;	[109]
*Lactobacillus* sp. & *Bacillus* sp.	Dietary supplementation at 0.75, 1.5, & 2.25 g kg^−1^	60 days	GP _WG_↑; FUP _FE_↑; DEA _PrA_↑; WQP _pH & Temp_↑ _and DO_↓	[111]
*Pediococcus acidilactici*	Dietary supplementation at 6×10^8^ CFU g^−1^	60 days	GP _FBW & SGR_↓; FUP _FCR & PER_↓; IP _Ig_↑; DEA _PrA_↑; HBP _TSP_↑; IRGE _TNF-α & SL_↓	[112]
*P. pentosaceus*	Dietary supplementation at 10^7^, 10^8^, & 10^9^ CFU g^−1^	45 days	GP _FBW, WG & SGR_↑; IP _ACA_ _& SL_↑; DEA _AmA, PrA, Trypsin, & Chymotrypsin_↑_,__and Lipase_→; HBP _WBC, Hb, Ht, & Alb_↑_,__and TC & Glu_→; IDR _*A. hydrophila*_↑	[113]
Primalac (*L. acidophilus*, *L. casei*, *E. faecium*, & *B. bifidium*)	Dietary supplementation at 1 & 1.5 g kg^−1^	8 weeks	GP _WG_↑_,__and_ _SGR_↓; FUP _FCR_↓_;_ HBP _WBC_↑_,__and RBC, Hb, Ht, MCV, MCH, & MCHC_↓; SR↑	[115]
Primalac	Dietary supplementation at 1 & 2 g kg^−1^	60 days	GP _FBW, WG, & SGR_↑; FUP _FCR_↓; DEA _AmA, lipase & protease_↑; HBP _TSP_↑, _and ALT, ALP, AST, TG, Glu, Cortisol, & TC_↓; IP _total_ _IgM, SL,_ _MPO, ACH50, SOD, CAT, GPx, & MDA_↑; SR↑	[114]
*B. subtilis*	Dietary supplementation at 4 × (10^6^ & 10^8^) CFU 100g^−1^	180 days	GP _WG & SGR_↑; FUP _FCE_↑_,__and_ _FCR_↓_,_ DNA: RNA↑	[151]

Symbol →, no change; ↑, increase; ↓, decrease versus controls.

ACA: Alternative complement activity; ACP: Acid phosphatase; ACH50: Alternative complement; Alb: Albumin; ALP: Alkaline phosphate; ALT: Alanine aminotransferase; AST: Aspartate aminotransferase; AmA: Amylase activity; CAT: Catalase; CD4^+^: Cluster of differentiation 4; CD8^+^: Cluster of differentiation 8; TC: Total cholesterol; CK: Creatine kinase; CP: Ceruloplasmin; DEA: Digestive enzyme activities; DO: Dissolved oxygen; FBW: Final body weight; FCE: Feed conversion efficiency; FCR: Feed conversion ratio; FUP: Feed utilization parameters; Glb: Globulin; Glu: Glucose; GP: Growth parameters; GPx: Glutathione peroxidase; GT: Glutamyl transpeptidase; GutM: Gut microbiota; Hb: Haemoglobin; HBP: Haemato-biochemical parameters; HSP: Heat shock protein; Ht: Haematocrit; IDR: Infectious disease resistance; Ig: Immunoglobulin; IL: Interleukin; iNOS: Inducible nitric oxide synthase; IP: Immunological parameters; IRGE: Immune related gene expression; MCH: Mean corpuscular haemoglobin; MCHC: Mean corpuscular haemoglobin concentration; MCV: Mean corpuscular volume; MDA: Melanodialdehyde; MPO: Myloperoxidase; NF: Nuclear Factor; PcA: Phagocytic activity; PER: Protein efficiency ratio; PrA: Protease activity; RB: Respiratory burst (NBT assay); RBC: Red blood cell; SBA: Serum bactericidal activity; SGR: Specific growth rate (%); SL: Serum lysozyme; SOD: Superoxide dismutase; SR: Survival rate; TAC: Total anti-oxidant capacity; TC: Total cholesterol; TG: Triglyceride; TGC: Thermal growth coefficient; TGF: Transforming growth factor; TNF: Tumor necrosis factor; TSP: Total serum protein; WBC: White blood cell; WG: Weight gain (%); WQP: Water quality parameters.

**Table 5:** Effects of probiotic supplementation on growth, feed utilizations, immunological and haemato-biochemical parameters and disease resistance in olive flounder (*Paralichthys olivaceus*).

Probiotics	Mode of administration and dosage	Duration	Effects on *P. olivaceus*	References
*Lactococcus lactis* BFE920	Dietary supplementation at 10^7^ CFU g^–1^ as oral vaccination	4 weeks	GP _WG_↑; FUP _FCR_↓; IRGE _CD4-1, CD4-2, CD8α,_ _T-bet_, _INF-γ, TLR5M, IL-1β, & IL-12p40_↑; Antigen-specific antibodies↑; IDR _*Edwardsiella tarda*_↑	[118]
*L. lactis* BFE920	Dietary supplementation at 10^7^ CFU g^–1^ as oral vaccination	4 weeks&3 Months	GP _WG_↑; FUP _FCR_↓; Antigen-specific antibodies↑; IRGE _CD4-1, CD4-2, CD8α,_ _& T-bet_↑; IDR _*Strepcoccus iniae*_↑	[117]
*L. lactis* BFE920 (LLBF) &*Ltobacillus plantarum* FGL0001 (LPFG)	Dietary supplementation at 10^7^ CFU g^–1^	30 days	IRGE _LLBF transcript FOXP3, IL-10, TGF- β1, CD18, & CD83_↑, _and LPFG_ _transcript IL-1β, CD18, T-bet, & INF-γ_ ↑; Gut permeability _LPFG_ ↑; Immune tone _30 days_; IDR _*E. tarda*_↑	[17]
*L. lactis* BFE920, *L. plantarum* FGL0001, & their combination	Dietary supplementation at 10^7^ CFU g^–1^	30 days	GP _WG_↑; IP _RB, PcA, & SMLA_↑; IRGE _TNF-α, IL-6, & IL-8_↑; IDR _*S. iniae*_↑	[35]
*L. plantarum* + *L. brevis* + *L. acidophilus* + *Bacillus subtilis* + *Saccharomyces cerevisiae*	Dietary probiotics mixtures in which each probiotic amount is 0.1%	12 weeks	GP _WG_↑; IP _RB, PcA, SL, & ACA_↑; HBP _ALT, AST, Glu, & TSP_↑	[119]
*L. plantarum*, *L. acidophilus*, *L. sakei*, *B. subtilis*, *S. cerevisiae*	Dietary supplementation of each probiotic at 2.42 × 10^8^ CFU g^–1^	8 weeks	GP _WG_↑; FUP _FER_↑; IP _RB & SL_↑; HBP _ALT, AST, TSP, & Glu_↑; SR _*L. plantarum*_↑	[120]
*L. fermentum*	Dietary supplementation at 0.5%	8 weeks	GP _FBW, WG, & SGR_→; FUP _FER & PER_→; HBP _ALT, AST, TC, TG & TSP_→; IDR _*S. iniae*_↑	[121]
*Bacillus* SJ-10	Dietary supplementation at 10^8^ CFU g^–1^	8 weeks	GP _FBW, WG & SGR_→; FUP _FCR_↓ _& PER_↑; IP _RB, SOD,__&__SL_↑_, and SAP & MPO_→; HBP _ALT, AST, Glu, TC, & TSP_→; IRGE _TNF-α, IL-1β, & IL-10_↑_, and IL-6, CD4-1, & CD4-2_→, PCI↑; IDR _*S. iniae*_↑; mVH→	[6]
*Bacillus* SJ-10	Dietary supplementation of heat-killed probiotic at 10^8^ CFU g^–1^	8 weeks	GP _FBW, WG & SGR_↑; FUP _FCR_↓ _& PER_↑; IP _SOD__&__SL_↑_, and RB, SAP & MPO_→; HBP _ALT, AST, Glu, TC, & TSP_→; IRGE _TNF-α, IL-6, & IL-1β_ ↑_, and IL-10_ →; IDR _*S. iniae*_↑; mVH→	[125]
*Bacillus* SJ-10	Dietary supplementation at 10^8^ CFU g^–1^	8 weeks	GP _WG & SGR_↑; FUP _FER_ _& PER_↑; IDR _*E. tarda*_↑	[116]
*Bacillus* sp. SJ-10 and *Lab. plantarum* KCCM 11322	Dietary supplementation at 10^8^ CFU g^–1^	8 weeks	GP _WG_↑; IP _SOD, SL, RB, MPO_↑; IDR _streptococcosis_↑	[43]
*B. subtilis*, *B. pumilus*, *B. licheniformis*	Dietary supplementation of each probiotic at 0.5%	8 weeks&5 days	GP _FBW_↑; FUP _FCR_↓ _& PER_↑; IP _RB & SOD_↑_, and SL, SAP, & MPO_→; SR _*B. subtilis*_↑; IDR _*S. iniae*_↑; WQP _Ammonia_↓ _by_ _*B. subtilis*_	[22]
*Bacillus* SJ-10, *Lab. plantarum*	Dietary supplementation at 10^8^ CFU g^–1^	8 weeks	DEA _AmA, Lipase, & Trypsin_↑, GutM _Proteobacteria, Actinobacteria, & Acidobacteria_↑; IRGE _BSJ-10 transcript TNF-α, IL-6, IL-1β, & IL-10_↑_, and CD4-1, INF-γ, CD4-2, CD-18, & CD-83_→; mVH and VH→; IDR _*S. iniae*_↑	[28]
*Bacillus* SJ-10	Dietary supplementation at 10^8^ CFU g^–1^	8 weeks	GP _FBW, WG & SGR_↑; FUP _FCR_↓ _& PER_↑, IRGE _BSJ-10 transcript TNF-α & IL-1β_↑, GutM _Richness & Diversity_↑; IDR _*S. iniae*_↑	[29]
*B. licheniformis* SK3927 + *B. amyloliquefaciens* SK4079 + *B. subtilis* SK4082 + *L. brevis* SK1751 + *L. plantarum* SK3494 + *S. cerevisiae* SK3587	Dietary supplementation at 10^8^ – 10^9^ CFU Kg^–1^	12 weeks	GP _FBW, WG, & SGR_→; FUP _FER, FCR & PER_→; IP _MPO, SL, & IgM_→_, and GPx_↑; SR→; GutM _*Ltobacillus, *Marinilactibacillus*,* & *Globicatella*_↑, _and *Biﬁdobacterium* & *Streptomyces*_↓; IRGE _TNF-α, IL-1β, & IL-6_↑	[126]
*Lac. lactis* BFE920	Dietary supplementation at 10^6^, 5 × 10^6^, 2.5 × 10^7^, 1.25 × 10^8^ CFU g^–1^ in laboratory & 2.5 × 10^7^ CFU g^–1^ in field experiment	2 weeks &3 months	_2 weeks_ IDR _*Streptococcus iniae*_↑; GP _WG_↑; FUP _FER_↑; IP _RB & MPO_↑; IRGE _IL-12 & INF-γ_ ↑; IDR _*S. iniae*_↑	[152]

Symbol →, no change; ↑, increase; ↓, decrease versus controls.

ACA: Alternative complement activity; ALT: Alanine aminotransferase; AmA: Amylase activity; AST: Aspartate aminotransferase; CD: Cluster of differentiation; DEA: Digestive enzyme activities; FBW: Final body weight; FCR: Feed conversion ratio; FER: Feed efficiency ratio; FOX: Forkhead box; FUP: Feed utilization parameters; Glu: Glucose; GP: Growth parameters; GPx: Glutathione peroxidase; GutM: Gut microbiota; Hb: Haemoglobin; HBP: Haemato-biochemical parameters; IDR: Infectious disease resistance; Ig: Immunoglobulin; IL: *Interleukin*; INF: Interferon; IP: Immunological parameters; IRGE: Immune related gene expression; MPO: Myloperoxidase; mVH: micro-Villous height; PcA: Phagocytic activity; PCI: Probiotic count in the intestine; PER: Protein efficiency ratio; RB: Respiratory burst (NBT assay); SAP: Serum antiprotease activity; SGR: Specific growth rate (%); SL: Serum lysozyme; SMLA: Skin mucus lysozyme activity; SOD: Superoxide dismutase; SR: Survival rate (%); TC: Total cholesterol; TG: Triglyceride; TGF: Transforming growth factor; TNF: Tumor necrosis factor; TRL5M: Membrane-anchored toll-like receptor 5; TSP: Total serum protein; WG: Weight gain (%).

**Table 6:** Effects of probiotic supplementation on growth, feed utilizations, immunological and haematobiochemical parameters and disease resistance in grass carp (Ctenopharyngodon idellus).

Probiotics	Mode of administration and dosage	Duration	Effects on *C. idellus*	References
*Bacillus licheniformis* FA6	Dietary supplementation at 10^5^ to 10^6^ CFU g^–1^	70 days	GP _WG & SGR_↑; IP _SOD_↑_,__and_ _MDA_↓; IRGE _IL-10, SOD, & CAT_↑_,__and_ _IL-1β, TNFα, TLR3, TLR7, & MyD88_↓; Intestinal morphology _VH & Muscle thickness_↑; IDR _*Aeromonas hydrophila*_↑	[128]
*B. subtilis* Ch9	Dietary supplementation at 2.4 × 10^7^ CFU g^–1^	42 days	GP _FBW, WG, & SGR_↑; IP _TAC, SOD, & CAT_↑_,__and MDA_↓; IRGE _IL-10, SOD, CAT, & GPx_↑_, and TNF-α, IL-8, & IL-1β_↓; IDR _*A. hydrophila*_↑	[129]
*B. subtilis* Ch9	Dietary supplementation at 10^7^ CFU g^–1^	8 weeks	GP _FBW, WG, & SGR_↑; HBP _ALT, HDL_↑, _and AST, TG, Alb, TP & TC_→; GutM↑; Fatty liver disease↓	[130]
*B. methylotrophicus* WM-1	Dietary supplementation at 10^3^, 10^5^ & 10^7^ CFU g^–1^	90 days	IP _SOD_↑; IDR _*A. hydrophila*_↑; SR↑	[131]
*Streptomyces amritsarensis* N1-32	Dietary supplementation at 10^7^ & 10^9^ CFU g^–1^	28 days	GP _WG_→; IP _SL, SOD, & ACP_↑; IRGE _IgM, TLR4, MyD88, Nrf2, & Keap1_↑_,__and TNF-α_→; IDR _*A. veronii*_↑	[132]
*Shewanella xiamenensis* A-1, 2, & *Aeromonas veronii* A-7	Dietary supplementation at 10^8^ CFU g^–1^	28 days	IP _RB, PcA, C3, & SL_↑; HBP _TSP, Alb, & Glb_↑; IRGE _IL-8, IL-1β, Lysozyme C, & TNF-α_↑; IDR _*A. hydrophila*_↑	[133]
*S. xiamenensis* A-1, *A. veronii* A-7 & *B. subtilis*	Dietary supplementation at 10^8^ CFU g^−1^	28 days	GutM _*Cetobacterium, *Citrobacter*, *Vibrio*, *Enterococcus*,* & *Streptococcus*_↑_,__and *Pseudomonas* & *Flavobacterium*_↓	[134]
*Pseudomonas stutzeri* F11	Water supplementation at 10^5^ CFU ml^−1^	9 days	WQP _Ammonia, Nitrite, & Total nitrogen_↓_,__and Nitrate_→; Water Microbiota _Bacteroidetes & Firmicutes_↑_,__and Proteobacteria, Actinobacteria, & Verrucomicrobia_↓	[135]
*B. licheniformis* BSK-4	Water supplementation at 10^8^ CFU m^*–*3^	18 days	WQP _Nitrite, Nitrate, & Total nitrogen_↓_,__and_ _Ammonia_↑; Water Microbiota _Proteobacteria & Firmicutes_↑_,__and Actinobacteria & Bacteroidetes_↓	[136]
*P. stutzeri* SC221-M & *B. cereus* BSC24	Water supplementation at 10^5^ & 3×10^5^ CFU ml^−1^	6 days	WQP _TDS, Ammonium, Nitrite, Total nitrogen, & COD_↓; Water Microbiota _Proteobacteria_↑_, and Bacteroidetes & Actinobacteria_↓	[137]
*B. natto* NT	Dietary supplementation at (1.8^,^ 3.73, 5.60, 7.47, & 9.33) × 10^9^ CFU 100 g^−1^	56 days	GP _FBW, WG & SGR_↑; FUP _FCR_↓; SR→; GRGE _miR-1a, miR-181a, miR-206, & miR-23a_↑_,__and_ _MyoG, MEF2C, & SRF_↓_, and miR-133a & miR-101a_→	[153]

Symbol →, no change; ↑, increase; ↓, decrease versus controls.

ACP: Acid phosphatase; ALT: Alanine aminotransferase; AST: Aspartate aminotransferase; Alb: Albumin, AmA: Amylase activity; C3: Complement C3; CAT: Catalase; COD: Chemical oxygen demand; DEA: Digestive enzyme activities; FBW: Final body weight; FCR: Feed conversion ratio; FUP: Feed utilization parameters; Glb: Globulin; GP: Growth parameters; GPx: Glutathione peroxidase; GRGE: Growth related gene expression; GutM: Gut microbiota; HDL: High-density lipoprotein; HBP: Haemato-biochemical parameters; IDR: Infectious disease resistance; Ig: Immunoglobulin; IL: *Interleukin*; IP: Immunological parameters; IRGE: Immune related gene expression; Keap1: Kelch-like ECH-associated protein 1 gene; LDL: Low-density lipoprotein; MDA: Malondialdehyde; MEF2C: Myocyte enhancer factor 2C; MPO: Myloperoxidase; MyD: Myeloid differentiation factor; MyoG: Myogenin; Nrf: Nuclear factor (erythroid-derived 2)-like 2 gene; PcA: Phagocytic activity; PrA: Protease activity; RB: Respiratory burst (NBT assay); SGR: Specific growth rate (%); SL: Serum lysozyme; SOD: Superoxide dismutase; SR: Survival rate; SRF: Serum response factor; TAC: Total anti-oxidant capacity; TC: Total cholesterols; TG: Triglyceride; TDS: Total dissolved solids; TLR: Toll like receptor; TNF: Tumor necrosis factor; TSP: Total serum protein; VH: Villous height; WG: Weight gain (%); WQP: Water quality parameters.

**Table 7:** Effects of probiotic supplementation on growth, feed utilizations, immunological and haematobiochemical parameters and disease resistance in rohu fish (*Labeo rohita*).

Probiotics	Mode of administration and dosage	Duration	Effects on *L. rohita*	References
*Bacillus subtilis* VSG1, *L. plantarum* VSG3, &*P. aeruginosa* VSG2	Dietary supplementation at (0.33, 0.5 & 1) × 10^8^ CFU g^–1^	60 days	GP _WG & SGR_↑; FUP _FCR_↓; IP _SL, ACA, PcA, RB, SOD, & IgM_↑; IDR _*A. hydrophila*_↑	[141]
*B. subtilis*, *L. lactis*, & *Saccharomyces cerevisiae*	Dietary supplementation at 10^11^ CFU Kg^–1^	30 days	GP _WG_↑; HBP _TSP, Glb, & RBC_↑_, and Glu, ACP, ALP, & WBC_↓; IP _RB, MPO, & IgM_↑; IRGE _HSP70_↑; Apoptosis↓	[142]
*B. methylotrophicus*, *B. amyloliquefaciens*, & *B. licheniformis*	Dietary supplementation at 10^7^ CFU g^–1^	60 days	GP _WG & SGR_↑; FUP _PER_↑_,__and_ _FCR_↓; HBP _Hb, RBC, WBC, TSP, Platelet, Glu, AST, & ALT_↑; IP _SL, ACA, PcA, RB, IgM, MPO, & SAP_↑; DEA _PrA, AmA, & Lipase_↑; IDR _*A. hydrophila*_↑	[143]
*B. aerophilus* KADR3	Dietary supplementation at 10^7^, 10^8^ & 10^9^ CFU g^–1^	3 & 6 weeks	HBP _TSP_↑; IP _SL, ACA, PcA, RB, SOD, & IgM_↑; IDR _*A. hydrophila*_↑	[20]
*B. amyloliquefaciens* CCF7	Dietary supplementation at 10^5^, 10^7^ & 10^9^ CFU g^–1^	70 & 28 days	HBP _Glb, Alb, & TSP_↑_,__and_ _AST & ALT_↓; IP _SOD, SL, CAT, & IgM_↑_,__and MDA_↓; IDR _*A. hydrophila*_↑	[144]
*B. amyloliquefaciens* COFCAU_P1	Dietary supplementation at 10^7^, 10^8^ & 10^9^ CFU g^–1^	30 days	HBP_Alb, Glb & TP_↑; IP _MPO, SOA & antiprotease_↑; IDR _*A. hydrophila*_↑; IRGE _TNF-α & IL-1β_↑; SR↑	[145]
*Geotrichum candidum* QAUGC01	Water supplementation at 10^9^ CFU L^–1^	70 days	GP _WG & SGR_↑; DEA _PrA, AmA, & Cellulase_↑; IDR _*Staphylococcus aureus*_↑; SR↑	[140]
*G. candidum* QAUGC01 & *B. cereus*	Dietary supplementation at 10^9^ CFU g^–1^	11 weeks	GP _FBW & SGR_↑; DEA _PrA, AmA, & Cellulase_↑; IDR _*A. hydrophila*_↑; GutM↑; SR↑	[138]
*Enterococcus faecium* QAUEF01, *E. faecium* QAUEF01 + *G. candidum* QAUGC01	Dietary supplementation at 10^9^ CFU g^–1^	90 days	GP _WG & SGR_↑; FUP _FCE_↑, _and_ _FCR_↓; HBP _RBC, WBC, Hb, & Lymphocyte_↑; DEA _PrA & Cellulase_↑; GutM↑	[146]
*S. cerevisiae*	Dietary supplementation at 0.5, 0.75 & 1%	60 days	GP _WG_↑; FUP _PER_↑_,__and_ _FCR_↓; HBP _Hb, RBC, WBC, ALT, & AST_↑; DEA _PrA & AmA_↑; IDR _*A. hydrophila*_↑	[147]
*G. candidum* QAUGC01	Dietary supplementation at 10^9^ CFU g^–1^	11 weeks	GP _WG_↑; HBP _Hb, RBC, WBC, Ht, & TSP_↑_,__and AST, ALT, TC, & TG_↓; IP _RB, SL, IgM, & PcA_↑; IRGE _HSP70_↑; DEA _PrA, AmA, & Cellulase_↑	[154]
*Lactobacillus* spp., *Streptococcus faecium*, *B. biﬁdum*, *B. subtilis*, *Sacchromyces* spp., *Torulopsis* & *Aspergillus oryzae*	Dietary supplementation at 1 & 1.5 g Kg^–1^	30 days	GP _FBW & SGR_↑; SR↑	[155]

Symbol →, no change; ↑, increase; ↓, decrease versus controls.

ACA: Alternative complement activity; ACP: Acid phosphatase; ALP: Alkaline phosphatase; ALT: Alanine aminotransferase; AmA: Amylase activity; AST: Aspartate aminotransferase; CAT: Catalase; DEA: Digestive enzyme activities; FCE: Feed conversion efficiency; FCR: Feed conversion ratio; FUP: Feed utilization parameters; Glb: Globulin; Glu: Glucose; GP: Growth parameters; GutM: Gut microbiota; Hb: Haemoglobin; HBP: Haemato-biochemical parameters; HSP70: Heat shock protein; IDR: Infectious disease resistance; IgM: Immunoglobulin M; IL: Interleukin; IP: Immunological parameters; IRGE: Immune related gene expression; MDA: Malondialdehyde; MPO: Myloperoxidase; PcA: Phagocytic activity; PER: Protein efficiency ratio; PrA: Protease activity; RB: Respiratory burst (NBT assay); RBC: Red blood cell; SAP: Serum antiprotease activity; SBA: Serum bactericidal activity; SGR: Specific growth rate (%); SL: Serum lysozyme; SOA: Superoxide anion; SOD: Superoxide dismutase; SR: Survival rate; TC: Total cholesterols; TG: Triglyceride; TNF: Tumor necrosis factor; TSP: Total serum protein; WBC: White blood cell; WG: Weight gain (%).
